# Effect of Remote Sensory Noise on Hand Function Post Stroke

**DOI:** 10.3389/fnhum.2014.00934

**Published:** 2014-11-17

**Authors:** Na Jin Seo, Marcella Lyn Kosmopoulos, Leah R. Enders, Pilwon Hur

**Affiliations:** ^1^Department of Industrial and Manufacturing Engineering, University of Wisconsin–Milwaukee, Milwaukee, WI, USA; ^2^Department of Occupational Science and Technology, University of Wisconsin–Milwaukee, Milwaukee, WI, USA; ^3^Department of Electrical Engineering and Computer Science, University of Wisconsin–Milwaukee, Milwaukee, WI, USA; ^4^Clinical & Translational Science Institute, Medical College of Wisconsin, Milwaukee, WI, USA; ^5^Department of Kinesiology, University of Wisconsin-Milwaukee, Milwaukee, WI, USA; ^6^Department of Mechanical Engineering, Texas A&M University, College Station, TX, USA

**Keywords:** sensory stimulation, sensory noise, stochastic resonance, stroke hand rehabilitation, hand function, tactile sensation

## Abstract

Hand motor impairment persists after stroke. Sensory inputs may facilitate recovery of motor function. This pilot study tested the effectiveness of tactile sensory noise in improving hand motor function in chronic stroke survivors with tactile sensory deficits, using a repeated measures design. Sensory noise in the form of subthreshold, white noise, mechanical vibration was applied to the wrist skin during motor tasks. Hand dexterity assessed by the Nine Hole Peg Test and the Box and Block Test and pinch strength significantly improved when the sensory noise was turned on compared with when it was turned off in chronic stroke survivors. The subthreshold sensory noise to the wrist appears to induce improvements in hand motor function possibly via neuronal connections in the sensoriomotor cortex. The approach of applying concomitant, unperceivable mechanical vibration to the wrist during hand motor tasks is easily adoptable for clinic use as well as unsupervised home use. This pilot study suggests a potential for a wristband-type assistive device to complement hand rehabilitation for stroke survivors with sensorimotor deficit.

## Introduction

Many strokes survivors suffer from persistent hand impairment (Wade et al., 1983; Parker et al., 1986; Trombly, 1989; Gray et al., 1990; Nakayama et al., 1994; Kamper et al., 2003). Hand impairment results in diminished vocational and self-care abilities (Hartman-Maeir et al., 2007), thereby affecting quality of life (Woodson, 2002). The hand impairment stems not only from muscle atrophy and altered supraspinal input to the muscles, but also from somatosensory deficits. It is well known that somatosensory feedback is a prerequisite for maintaining and regaining optimal motor control (Pearson, 2000; Edgerton et al., 2001; Perez et al., 2003). Tactile signals convey information about skin pressure, stretch, and vibration (Roudaut et al., 2012), while proprioceptive signals convey information about the state of the limb (Sainburg et al., 1995). For hand grip, tactile feedback along with proprioceptive feedback provides information about the shape, size, and texture of objects and whether a grasped object is slipping from the grip (Johansson and Flanagan, 2009). Impaired tactile sensation of the fingers via local anesthesia in healthy adults (Johansson and Westling, 1984; Augurelle et al., 2003; Monzee et al., 2003) severely disturbs the ability to approximate the grip force required to grasp an object, manipulate objects, and sustain grasp. In addition, tactile sensory input appears to influence maximal grip strength, which used to be thought to reflect pure motor capacity (Enders and Seo, 2011; Seo et al., 2011), with decreased maximum pinch grip strength after local anesthesia (Augurelle et al., 2003). Chronic sensory deficits following nerve compression (Keith et al., 2009) or stroke (Blennerhassett et al., 2006, 2007) lead to clumsiness and a learned non-use of the affected hand (Carey et al., 1993).

Post-stroke somatosensory deficits are quite prevalent (Carey, 1995; Turton and Butler, 2001; Connell et al., 2008), with 50–85% of stroke survivors exhibiting them (Kim and Choi-Kwon, 1996; Carey and Matyas, 2005, 2011). Reduced afferent inputs can result in cortical reorganization that is not limited to the somatosensory cortex, but extends to the motor cortex (Weiss et al., 2004), suggesting that sensory input is integral to the preservation of sensorimotor cortical representations and limb function following stroke. Given the important role of sensory input in motor control, it is not surprising that post-stroke motor recovery is significantly associated with the extent of tactile and proprioceptive sensory deficit (Tyson et al., 2008; Meyer et al., 2014).

The need for intact sensory input preceding motor rehabilitation is clear. As such, a few methods to influence the sensory system have been developed. They include sensory discrimination training, passive sensory stimulation, temporary deafferentation, and sensory noise. Sensory discrimination training involves patients’ repeated practice to distinguish textures, localize tactile stimulus, and detect body positions (Carey and Matyas, 2005; Carey, 2006). The idea is that improved perceptual somatosensation in both tactility and proprioception may lead to improved somatosensory feedback needed for dexterous motor function. Sensory discrimination training alone was not found to result in significant improvement in tactile or proprioceptive sensation in meta-analysis (Schabrun and Hillier, 2009) or better upper limb function compared to conventional occupational therapy in a randomized controlled study (Chanubol et al., 2012). On the other hand, passive sensory stimulation involves application of electrical (Wu et al., 2006; Celnik et al., 2007; Conforto et al., 2007, 2010), magnetic (Tegenthoff et al., 2005), or tactile stimulation (Smith et al., 2009) to sensory nerves for up to 2 h. Passive sensory stimulation intends to activate the nerve fibers that transmit somatosensory input originating from peripheral receptors critical for sensorimotor performance, thereby possibly eliciting cortical reorganization in the somatosensory as well as in the primary motor cortex via direct anatomic projections from the somatosensory cortex (Wu et al., 2006). Meta-analysis suggests some evidence to support the use of passive sensory stimulation to improve hand dexterity and grip strength in stroke survivors (Schabrun and Hillier, 2009). The third method involves temporary deafferentation via anesthesia of the affected forearm or the contralateral hand to result in better sensory and motor performance at least temporarily, possibly by decreasing inhibitory drive to the affected hand’s sensorimotor areas (Voller et al., 2006; Weiss et al., 2011; Sens et al., 2012).

The sensory noise method involves application of a small level of mechanical vibration to the skin to result in immediate improvement in sensorimotor function (Collins et al., 1996b, 2003). The concept of stochastic resonance, in which the addition of noise improves signal detection and feedback-controlled system performance, has been demonstrated theoretically (Duan et al., 2013) as well as in myriads of biological systems (Wiesenfeld and Moss, 1995; Collins et al., 1996a,b; Moss et al., 2004; Fertonani et al., 2011). For example, subthreshold vibrotactile noise applied to the feet has been shown to improve foot tactile sensation in stroke survivors and healthy young and old adults (Liu et al., 2002; Wells et al., 2005), subsequently reducing postural sway in stroke survivors, diabetic patients, and healthy adults (Priplata et al., 2002, 2006) and also reducing gait variability in healthy old adults (Galica et al., 2009). For hand function, subthreshold vibrotactile noise directly applied to the index fingertip has been shown to immediately improve fingertip tactile sensation in stroke survivors (Liu et al., 2002) and healthy adults (Kurita et al., 2013) and reduce the amount of excess grip force for lifting an object, indicating more efficient grip in healthy adults (Kurita et al., 2013).

Application of noise to the forearm, although not directly to the hand, has also been shown to improve hand sensorimotor function by shortening reaction time to hand tactile stimuli in healthy adults (Hur et al., 2014). Such a remote effect (noise applied to the forearm having an effect on the hand tactile perception) has the practical implication of strategically placing a noise generator off the hand in order to expose the entire hand skin for tactile stimuli during dexterous manual tasks. Similar improvements were seen in stroke survivors where subthreshold vibrotactile noise applied to the wrist or dorsum of the hand improved thumb and index finger touch sensation (Enders et al., 2013).

While presenting great potential to meet the need of stroke survivors with sensorimotor deficits in the hand, application of subthreshold vibrotactile noise off the hand has not been examined for its efficacy in improving stroke survivors’ hand motor function and dexterity. The objective of this study was to evaluate the effectiveness of subthreshold vibrotactile noise applied to the wrist in improving hand motor function for stroke survivors with tactile sensory deficits. The hypothesis was that use of subthreshold vibrotactile noise at the wrist would enhance hand motor function in stroke survivors. In particular, hand dexterity and maximum pinch grip strength were hypothesized to improve with the sensory noise, because sensory feedback is critical for dexterous hand movement (Johansson and Westling, 1984; Augurelle et al., 2003; Monzee et al., 2003; Keith et al., 2009), and sensory input helps increase maximum pinch strength as described earlier.

## Materials and Methods

### Subjects

Ten chronic stroke survivors (>6 months post stroke) with tactile sensory deficits were recruited for this study. Stroke survivors with a Semmes-Weinstein monofilament score >2.83 on either the thumb tip or the index fingertip were defined as having tactile sensory deficits (Cooper and Canyock, 2013). Individual subjects’ demographic information including age, gender, time since stroke, and motor impairment level is shown in Table 1. All signed a written consent form and followed protocol approved by the Institutional Review Board.

**Table 1:** **Participant characteristics**.

Subject	Age	Gender	Paretic hand	Time since stroke (years)	Chedoke (/7)	Fugl-Meyer (/24)
V01	62	M	Left	14	7	22
V02	62	M	Left	7	6	16
V03	63	F	Left	10	5	16
V04	53	F	Left	5	7	24
V05	68	F	Left	9	2	2
V06	60	M	Left	9	5	22
V07	56	M	Right	5	6	14
V08	82	M	Right	2	6	23
V09	67	M	Left	2	7	24
V10	61	M	Right	12	7	24

*Chedoke: the Hand Section of the Chedoke-McMaster stroke assessment scale; Fugl-Meyer: upper extremity portion of the Fugl-Meyer assessment*.

### Procedure

Stroke survivors’ paretic hand motor function was compared with and without subthreshold vibrotactile noise to the wrist. A set of hand function tests was repeated in four blocks, without noise for blocks one and four and with noise for blocks two and three. Learning effects were accounted by providing a practice block prior to data collection.

#### Vibrotactile noise

**Figure 1:**
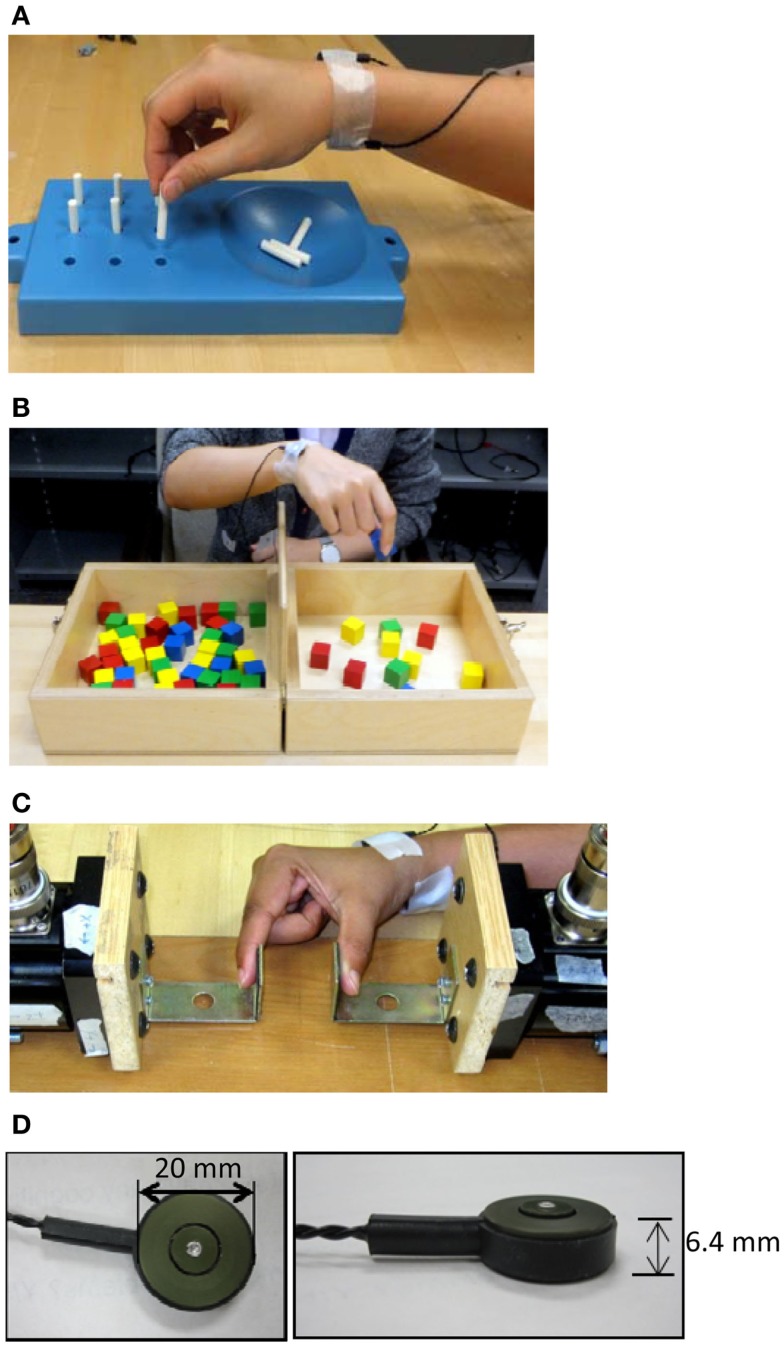
**The subjects performed hand motor tasks including the Nine Hole Peg Test (A), Box and Block Test (B), and maximum pinch grip (C), while the noise generators (D) placed on the wrist were turned on or off**.

Vibrotactile noise was applied using two C-3 tactors (Engineering Acoustics, Inc., Casselberry, FL, USA) attached to the volar and dorsal wrist of the paretic arm using adhesive tapes (Figure 1). White noise signals low-pass filtered at 500 Hz drove the tactors, as in the previous study (Enders et al., 2013). The tactors were attached on the wrist to minimize interruption with manual tasks, while still affecting finger tactile sensation as shown in the previous study (Enders et al., 2013). The intensity of the vibrotactile noise was set to 60% of the sensory threshold found at the beginning of testing. That intensity was used as it is approximately the optimal noise level to affect the sensory system per the literature (Wells et al., 2005) and our previous study with that noise intensity to the wrist has shown to improve fingertip tactile sensation in chronic stroke survivors (Enders et al., 2013). The vibrotactile noise generators were attached for the duration of the testing and were turned off or on at the beginning of each block depending on the noise condition. Subjects were blinded to the noise, as they could not feel the noise.

#### Hand motor function tests

Hand dexterity and pinch grip strength constituted the main outcome measures for the hand motor function. Hand manual dexterity was assessed using the Nine Hole Peg Test (NHPT) and the Box and Block Test (BBT) (Figures 1A,B). These tests provide reliable measurement of manual dexterity (Falconer et al., 1991; Desrosiers et al., 1994; Chen et al., 2009). The NHPT and BBT were administered according to the literature (Mathiowetz et al., 1985; Oxford Grice et al., 2003). Pinch grip strength was assessed as maximum normal force recorded during maximum voluntary precision pinch grip using the thumb and index finger (Figure 1C). During the maximum pinch grip, grip force deviation, the extent to which the digit force deviated from the direction normal to the grip surface, was also quantified as the arctangent of shear force to normal force ratio, as this deviation was shown to be excessive post stroke, contributing to impaired grip (Seo et al., 2010).

In addition to the hand motor function, wrist motor function was assessed using the active range of motion (ROM), in case the increased motor output with the sensory noise to the wrist extends beyond the hand. The active ROM of the wrist was measured using a digital goniometer while the subject voluntarily and maximally flexed and extended their wrist. To replicate the previous finding of finger sensory enhancement with the vibrotactile noise to the wrist (Enders et al., 2013), the monofilament test for the thumb and index fingertips was performed at the end of each set of hand motor function tests. The monofilament test was performed following the literature (Bell-Krotoski et al., 1993). Rest breaks of a 2 min minimum were provided between each test and of a 5 min minimum between blocks. More rest breaks were given if requested by the subject.

### Data analysis

For the main analysis, the Kruskal–Wallis test was used on the multivariate data to test if the noise (with vs. without) significantly affected the main outcome measures – the NHPT time, BBT score, and pinch grip strength. For the secondary analysis, three additional Kruskal–Wallis tests were performed to examine the effect of noise on the grip force deviation, the wrist ROM and the monofilament score, separately. In addition, responsiveness of the individual main outcome measures to the noise intervention and correlations among the noise-induced changes in the three main outcome measures were examined using the standardized response means (Cohen, 1988) and Spearman rank correlation tests (Portney and Watkins, 2009), respectively.

## Results

**Figure 2:**
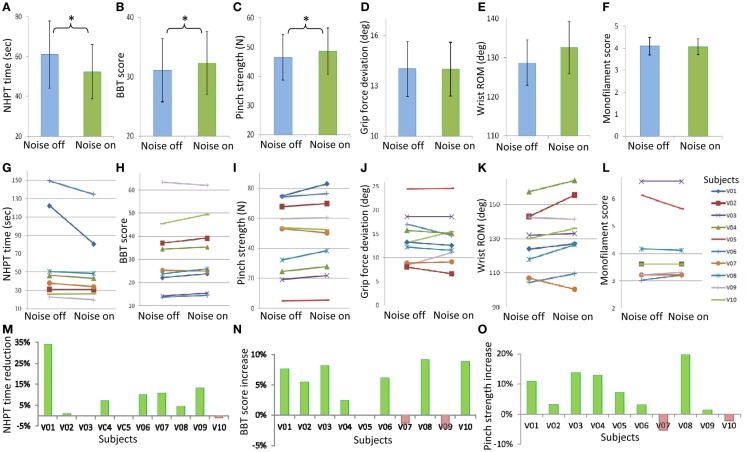
**Mean ± standard error values for the time to complete the Nine Hole Peg Test (NHPT time, A), the number of blocks subjects could move during the Box and Block Test (BBT score, B), pinch grip strength (C), grip force deviation during pinch grip (D), wrist active range of motion (ROM, E), and monofilament score (F) without and with the remote sensory noise are shown on the top row**. Statistical significance is noted with stars. Individual subjects’ values without and with the remote sensory noise are shown on the middle row **(G–L)**. For the three main outcome measures with statistical significance, percent changes with the remote sensory noise (compared to without noise) for individual subjects are shown on the third row **(M–O)**, with a favorable change having a positive sign. The mean values of the two blocks with noise off and of the two blocks with noise on are shown.

Subthreshold vibrotactile noise to the wrist significantly improved stroke survivors’ hand dexterity and strength (*p* = 0.037, Figure 2). Improvement in hand dexterity and strength was shown by decreased NHPT time and increased BBT score, and increased maximum pinch strength, respectively. With the remote subthreshold vibrotactile noise, subjects were able to shorten the time to complete the NHPT, on average, by 14%, compared to without the noise (Figure 2A). Seven stroke survivors shortened the NHPT time, while one subject showed lengthened NHPT time with the noise (Figures 2G,M). Two subjects could not move any peg in 2 min with or without the sensory noise. With the remote subthreshold vibrotactile noise, subjects were also able to move greater more number of blocks in the BBT, on average, by 4% (Figure 2B). Seven stroke survivors increased the BBT score, on average by 7%, while two subjects showed decreased BBT scores, on average by 2% (Figures 2H,N). One subject could not move any block in a minute with or without the noise. The average pinch strength increased by 5% with the noise (Figure 2C). Eight stroke survivors increased their maximum pinch grip strength with the noise, on average by 9%, whereas two subjects showed, on average, 4% decreased maximum pinch grip strength (Figures 2I,O). Responsiveness was similar among the three main outcome measures, with the standardized response means of 0.53, 0.74, and 0.67 for the NPHT time, BBT score, and maximum pinch grip strength, respectively, showing moderate responsiveness (Cohen, 1988; Lin et al., 2010). The correlations among the noise-induced changes in the three main outcome measures in individual patients ranged from 0.35 to 0.40, indicating fair correlation (Portney and Watkins, 2009; Lin et al., 2010). For the other three measures, the mean grip force deviation decreased (Figure 2D), the mean wrist active ROM increased (Figure 2E), and the mean smallest monofilament size that they could perceive decreased (Figure 2F) with subthreshold vibrotactile noise, but without statistical significance (*p* > 0.05 for all three measures).

## Discussion

### Improvement in hand dexterity and strength with the sensory noise

In this pilot study, remote sensory noise enhanced the hand motor function for chronic stroke survivors with tactile sensory deficits, as seen by improved NHPT, BBT, and maximum pinch grip strength with subthreshold vibrotactile noise applied to the wrist. Moderate responsiveness was observed for all three main outcome measures. These improvements support the hypothesis that remote sensory noise facilitates hand dexterity and strength. While facilitating dexterity and maximum pinch grip strength, the remote sensory noise did not appear to affect the active ROM of the wrist, suggesting that the wrist sensory noise influenced coordination and activation of muscles located within the hand, but not those of the forearm muscles. Such specificity may suggest involvement of direct connections between the somatosensory and motor cortices (Jones et al., 1978; Strick and Preston, 1982; Stepniewska et al., 1993; Wu and Kaas, 2003), specifically between the wrist somatosensory area and the finger motor area. Such cortical connections may differ among patients depending on the lesion and rehabilitation therapies they received resulting in different cortical reorganizations, which may explain only fair correlations among the noise-induced changes in the three main outcome measures in individual patients. The lack of the noise effect on grip force deviation may be because during high force exertions, mechanoreceptors on the fingertip pads are overloaded with high tactile pressure, and thus control of grip force deviation may not rely on sensory feedback-based motor control. A similar observation regarding the lack of an effect of tactile sensory intactness on pinch force reproduction at high force levels as opposed to a low force level (De Serres and Fang, 2004) supports this postulation.

Contrary to the previous study (Enders et al., 2013), finger sensation did not change with subthreshold vibrotactile noise to the wrist in this pilot study. Yet, improvements in dexterity and strength were observed. This is similar to previous studies using temporary deafferentation that observed no significant correlation between the extent of improvement in tactile sensation and the extent of improvement in motor performance (Weiss et al., 2011; Sens et al., 2012). The previous studies offered the possibility that the motor system and perceptual somatosensation could be independently influenced by the sensory manipulation. Another potential explanation for no significant change in the monofilament score observed in this study may be adaptation to noise. The monofilament test was always performed last, after all motor tests were completed in each block. By the time the monofilament test was administered, subjects were exposed to the noise for approximately 15–20 min continuously (in each of blocks 2 and 3) and may have been acclimatized to the noise. Sensory adaptation is a well-known phenomenon where the skin loses sensitivity in the area exposed to prolonged stimulation (Berglund and Berglund, 1970; Dinse and Merzenich, 2002). Such adaptation is mediated through reduced responsiveness of both first-order mechanoreceptive afferents (Lundstrom and Johansson, 1986; Bensmaia et al., 2005) and central nervous system neurons (O’Mara et al., 1988). Thus, prolonged exposure to the noise could have incurred sensory adaptation. Alternatively, adaptation could have occurred at the perceptual level, as opposed to the afferent sensory nerves. Improvement in the monofilament score was seen when the remote noise was turned on and off immediately before and after sensory testing in the previous study (Enders et al., 2013). Therefore, in this pilot study, any enhancement in the finger sensation could have dissipated by the time the monofilament test was performed, or alternatively, the perceptual adaptation could have occurred after the long exposure to the sensory noise.

A number of previous studies have also shown motor improvement immediately following application of sensory noise or sensory stimulation not only in healthy adults but also in stroke survivors with motor deficits (Priplata et al., 2002, 2006; Collins et al., 2003; Galica et al., 2009; Smith et al., 2009; Kurita et al., 2013). These past findings, together with this pilot study’s findings, collectively support the hypothesis that interventions involving the somatosensory system may facilitate motor recovery in stroke survivors.

### Unique characteristics of the sensory noise

The present study applied sensory noise simultaneously with targeted motor tasks to induce improvement in hand dexterity and strength. The use of noise during targeted motor tasks for instant effects differentiates this method from others that apply sensory stimulation for up to 2 h at a time to prime the sensory system prior to targeted motor tasks (Tegenthoff et al., 2005; Sawaki et al., 2006; Smith et al., 2009) or sensory discrimination training that has to be conducted separately from targeted motor tasks (Carey and Matyas, 2005; Carey, 2006; Chanubol et al., 2012). The instantaneous influence without the need to be exposed for an extended period of time may provide a practical benefit for using this technique. This aspect of the technique is similar to functional neuromuscular electrical stimulation (Santos et al., 2006), except that functional neuromuscular electrical stimulation intends to directly augment contraction of muscles that are needed for targeted motor tasks with a high stimulation intensity, whereas sensory noise intends to influence the sensory system thereby indirectly influencing the motor system using a low stimulation intensity, as described below.

The present method used subthreshold sensory noise to induce improvement in hand dexterity and strength. It is different from other techniques that used suprathreshold sensory stimulation, sometimes strong enough to cause paresthesia (Conforto et al., 2002, 2007, 2010; Sawaki et al., 2006; Wu et al., 2006; Celnik et al., 2007). Although not perceivable, subthreshold sensory stimuli have been shown to cause perceptual and behavioral changes (Watanabe et al., 2001). Direct application of subthreshold noise to a tactile signal helps detection of the signal, whereas suprathreshold noise directly added to the tactile signal swamps the signal and interferes with signal detection in the framework of stochastic resonance (Collins et al., 1997; Wells et al., 2005). Exposure to tactile stimulation far above the sensory threshold on the fingertip pad impaired tactile sensation on that skin area, whereas stimulation approximately at the sensory threshold improved tactile sensation (Collins et al., 1997; Ragert et al., 2008). The optimal level of direct noise for improving tactile sensitivity appears to be 33–67% of the sensory threshold (Wells et al., 2005), to which the remote noise intensity used in the present study belongs. In summary, although unperceivable, subthreshold sensory noise appears to be capable of influencing the human sensorimotor system.

White noise was used in the present study. As opposed to constant frequency stimulation, white noise may reduce sensory adaptation and enhance the effect of sensory stimulation (McDonnell and Abbott, 2009; Fertonani et al., 2011). Exposure to temporally non-uniform tactile stimulation improved tactile sensation, whereas constant frequency tactile stimulation impaired tactile sensation on the stimulated skin area (Ragert et al., 2008). Random frequency transcutaneous electrical stimulation delivered better sensory stimulation and pain management compared to conventional constant frequency stimulation (Bloodworth et al., 2004). Temporally, non-uniform electrical nerve stimulation modulated strength of a spinal circuit, whereas constant frequency stimulation did not (Perez et al., 2003). Even with the white noise, however, sensory or perceptual adaptation could have occurred after 15–20 min of exposure in the present study as discussed earlier.

The last unique feature of this study is that the sensory noise was applied to the wrist, with resulting improvements in hand dexterity and strength. Spreading effects of sensory manipulation have been shown in the past. For instance, subthreshold vibrotactile noise to the wrist resulted in improved touch sensation on the fingers in stroke survivors (Enders et al., 2013). Constant frequency tactile stimulation on the index finger resulted in impaired tactile sensation not only for the index finger but also for the middle finger in healthy adults (Ragert et al., 2008). Furthermore, sensory manipulation resulted in changes in the sensorimotor function of a remote body part. For instance, forearm anesthesia resulted in not only sensory loss in the forearm, but also improved sensorimotor function of the hand, in healthy adults (Bjorkman et al., 2004) as well as in stroke survivors (Weiss et al., 2011; Sens et al., 2012). Subthreshold vibrotactile noise to the forearm resulted in early reaction time in response to hand tactile stimuli in healthy adults (Hur et al., 2014). The mechanism of the spreading effects of the tactile stimulation to sensorimotor function of other body parts does not likely involve propagation of tactile stimulation along the skin, since mechanical vibration loses its power by more than 90% when it travels 1–2 cm on the skin (Manfredi et al., 2012; Kurita et al., 2013). In addition, since the vibrotactile noise intensity was very small, far below the level the person could feel, change in attention or stimulation of tendons or other tissues at the wrist is unlikely to have occurred to induce effects on the hand. Instead, it is possible that sensory manipulation simply unmasks pre-existing synaptic connections within the central nervous system (Merzenich et al., 1983; Hidaka et al., 2000; Manjarrez et al., 2003; Bjorkman et al., 2004; Ragert et al., 2008). For instance, noise applied to the arterial baroreceptor in the neck optimized the baroreflex response to pressure signals detected by the cardiopulmonary baroreceptor in the heart (Hidaka et al., 2000), showing that the noise and signal at two different bodily locations can be integrated within the central nervous system and that the noise can affect another circuitry via neuronal connections.

### Clinical implication

The focus of stroke rehabilitation is to regain or improve function. For stroke survivors, improving function could mean an increased ability to perform activities of daily living. Manual dexterity has been shown to be indicative of functional independence. Noise-induced improvements seen in reliable measures such as the NHPT, BBT, and hand strength in this pilot study indicate the possibility of increased functional independence.

The features of our approach enable the potentially easy adoption of subthreshold sensory noise for home or clinic use. Our approach applied an unperceivable, minute level of vibration to the wrist, concomitantly during targeted motor tasks, with instant effects on hand motor function. Simple mechanical vibration can be produced with low-cost devices and fewer safety concerns, compared with deafferentation via anesthesia, constant current electrical stimulation, or transcranial magnetic stimulation, which are not readily accessible and have greater safety risks. Unperceivable, minute vibration does not cause discomfort, pain, or paresthesia, and is thus more patient-friendly. Application of noise to the wrist, as opposed to the fingers (Liu et al., 2002; Kurita et al., 2013), prevents the noise-generating device from interfering with object manipulation and dexterous hand movement and also exposes the entire hand for tactile stimuli. The approach can be used concomitantly during therapies, without the need to wait for hours. Relatively minimal safety concerns, discomfort, and time demand, together with low-cost and potential for unsupervised use directly by patients, make this approach highly practical.

The improvements reported in this pilot study may be small, although they are statistically significant. These improvements were obtained instantaneously, and repeated use with therapy may result in greater clinical impact by allowing practices in sensorimotor integration and providing intensity needed for recovery (Kwakkel, 2006). In addition, use of this sensory noise technique early during the acute rehabilitation phase may yield much greater benefits by stimulating the sensorimotor system during the period in which rapid neural reorganization and regeneration occur. Further studies are needed to strengthen the preliminary findings of this pilot study.

Use of this approach during therapy may be designed with consideration of possible sensory adaptation. Therapy with continuous noise longer than 15–20 min may not yield additional benefits of using the noise to improve sensory perception. Thus a few minute break may be taken before continuing another round of therapy for 15–20 min to recover from sensory adaptation (Berglund and Berglund, 1970). Alternatively, the noise can be turned on only during active tasks. When the noise was turned on only for active tasks and turned off between tasks, no residual effect of the noise was seen within a two-hour period (Enders et al., 2013).

## Conclusion

In this pilot study, hand dexterity and strength improved with subthreshold vibrotactile noise at the wrist in chronic stroke survivors with tactile sensory deficits. The noise-induced improvements in hand motor function may have been mediated by cortical interneuronal connections from the wrist somatosensory area to the finger motor area. The approach of applying concomitant, unperceivable mechanical vibration to the wrist during hand motor tasks is easily adoptable for clinic use as well as unsupervised home use. This pilot study suggests the potential for developing an assistive device worn at the wrist, applying subthreshold vibrotactile noise to enhance hand motor function. Such a device would be placed remotely from the fingers and palm so as not to interfere with object manipulation or dexterous hand function and to allow the hand to receive relevant tactile stimuli. Such an assistive device or sensory orthosis may complement hand rehabilitation for patients with stroke with sensorimotor deficit, and thus, lead to increased functional independence and enhanced quality of life.

## Conflict of Interest Statement

There is a pending patent for a wearable device for improving tactile sensitivity, involving use of the sensory noise, similarly with the present manuscript. Na Jin Seo and Leah R. Enders are listed as inventors of this pending patent.
